# Coping in the Emergency Medical Services: Associations With the Personnel’s Stress, Self-Efficacy, Job Satisfaction, and Health

**DOI:** 10.32872/cpe.6133

**Published:** 2022-03-31

**Authors:** Roberto Rojas, Maxi Hickmann, Svenja Wolf, Iris-Tatjana Kolassa, Alexander Behnke

**Affiliations:** 1 University Psychotherapeutic Outpatient Clinic, Institute of Psychology and Education, Ulm University, Ulm, Germany; 2 Clinical and Biological Psychology, Institute of Psychology and Education, Ulm University, Ulm, Germany; Philipps-University of Marburg, Marburg, Germany

**Keywords:** Emergency Medical Services, coping strategies, stress, job satisfaction, work-related self-efficacy

## Abstract

**Background:**

Emergency Medical Services personnel (EMSP) are recurrently exposed to chronic and traumatic stressors in their occupation. Effective coping with occupational stressors plays a key role in enabling their health and overall well-being. In this study, we examined the habitual use of coping strategies in EMSP and analyzed associations of coping with the personnel’s health and well-being.

**Method:**

A total of N = 106 German Red Cross EMSP participated in a cross-sectional survey involving standardized questionnaires to report habitual use of different coping strategies (using the Brief-COPE), their work-related stress, work-related self-efficacy, job satisfaction, as well as mental and physical stress symptoms.

**Results:**

A confirmatory factor analysis corroborated seven coping factors which have been identified in a previous study among Italian emergency workers. Correlation analyses indicated the coping factor “self-criticism” is associated with more work-related stress, lower job satisfaction, and higher depressive, posttraumatic, and physical stress symptoms. Although commonly viewed as adaptive coping, the coping factors “support/venting”, “active coping/planning”, “humor”, “religion”, and “positive reappraisal” were not related to health and well-being in EMSP. Exploratory correlation analyses suggested that only “acceptance” was linked to better well-being and self-efficacy in EMSP.

**Conclusion:**

Our results emphasize the need for in-depth investigation of adaptive coping in EMSP to advance occupation-specific prevention measures.

## Highlights

- Previously reported seven factor structure of BriefCOPE was confirmed in German EMS personnel.
- Adaptive coping factors (e.g., support/venting) are not linked to better health and well-being.
- Self-criticism correlates with lower job satisfaction, higher stress, and more stress symptoms.
- Acceptance is associated with less stress symptoms and higher self-efficacy.

Emergency Medical Services personnel (EMSP) are recurrently confronted with traumatic events during medical rescue missions and undergo adverse working conditions such as shiftwork, time pressure, insufficient sleep, and social conflicts (Donnelly & Siebert, 2009; Karutz et al., 2013; Sterud et al., 2006). These factors pose a high emotional stress on EMSP (Johnson et al., 2005; Karrasch et al., 2020; Schmid et al., 2008), which can compromise their job satisfaction (Boudreaux et al., 1997; Portero de la Cruz et al., 2020; Sterud et al., 2011) and may trigger mental health problems, including depression, posttraumatic stress disorder (PTSD), and alcohol abuse (Berger et al., 2012; Kleim & Westphal, 2011; Petrie et al., 2018; Sterud et al., 2006; S. L. Wagner et al., 2020) as well as physical health problems (Aasa et al., 2005; Bentley & Levine, 2016; Friedenberg et al., 2022; Hegg-Deloye et al., 2014).

To maintain their health and work capacity, EMSP are required to employ effective strategies to cope with chronic stress and recurrent exposure to traumatic events on duty (Arble & Arnetz, 2017; Karrasch et al., 2020). *Coping* is defined as a person’s effort to deal with external or internal demands that are perceived as stressful or possibly exceed the individual’s resources (Lazarus & Folkman, 1984). Research has described various strategies to cope with stress. Some of them such as social support seeking, acceptance, and positive reappraisal are viewed as adaptive in reducing stress and benefiting health and well-being (Holton et al., 2016; Moritz et al., 2016). Conversely, strategies involving self-criticism, denial, dissociation, and avoidance are viewed as maladaptive for stress management and can lead to impaired health and well-being (Holton et al., 2016; Prati & Pietrantoni, 2009).

In the context of their work, EMSP and other frontline workers are confronted with high emotional demands and physical stressors due to shift work, time pressure, high responsibility, and recurrent traumatic event exposure. As a result, EMSP may find certain coping strategies not helpful in handling their work-related demands, although in other contexts, the same strategies may be highly adaptive, and vice versa. In this line, growing evidence shows that coping strategies may differ in their actual adaptiveness depending on the context of their application (Cheng et al., 2014; Folkman & Moskowitz, 2004; Levy-Gigi et al., 2016).

## “Maladaptive” Coping in EMSP

There is consistent evidence that “maladaptive” coping strategies are linked to poorer well-being and health in EMSP. *Self-criticism* is linked to more burnout, compassion fatigue, depression, and PTSD symptoms, and lower compassion satisfaction (Boland et al., 2019; Boudreaux et al., 1997; Cicognani et al., 2009; Kirby et al., 2011; Prati et al., 2011). Furthermore, avoidant coping such as *substance (ab)use* and *denial* was linked to poorer mental health outcomes in the long-term such as elevated PTSD symptoms (Arble & Arnetz, 2017; Cicognani et al., 2009; Kerai et al., 2017; Kirby et al., 2011; LeBlanc et al., 2011; Portero de la Cruz et al., 2020; Regehr et al., 2002). Despite negative consequences, EMSP engage in avoidant coping because these strategies allow to instantly alleviate emotional strain (Levy-Gigi et al., 2016; Regehr et al., 2002). For example, it was shown that EMSP use emotional avoidance after critical mission incidents (Figley, 2008).

## “Adaptive” Coping in EMSP

Previous studies reported that coping strategies, which are assumed adaptive in the general population, show inconsistent or even negative associations with the well-being and health of EMSP (Cicognani et al., 2009; Prati et al., 2011; Raynor & Hicks, 2019). Upon exposure to stressful events, EMSP may profit from *social support* to receive emotional support and relief (Alexander & Klein, 2001; ALmutairi & El Mahalli, 2020; Boland et al., 2019; Donnelly & Siebert, 2009). In EMSP, social support has been associated with lower risk of depressive, burnout, and trauma-related symptoms (Boland et al., 2019; Essex & Scott, 2008; Feldman et al., 2021; Fjeldheim et al., 2014; Guilaran et al., 2018; Prati & Pietrantoni, 2010; Wild et al., 2016). However, other studies found that social support did not moderate the negative influence of stressful mission experiences on PTSD symptoms (C.-M. Chang et al., 2008). Higher social support was also linked to burnout and compassion fatigue among EMSP (Cicognani et al., 2009; Prati et al., 2011).

Moreover, EMSP may cope actively with stress through focusing on the next step in planning and actively solving problems (Boland et al., 2019; Regehr et al., 2002). *Active coping/planning* was associated with lower stress levels (Brown et al., 2002; Jamal et al., 2017) and stronger posttraumatic growth (Kirby et al., 2011) in EMSP. However, Folkman and Moskowitz (2004) theorized that the effectivity of active coping depends on the controllability of stressors. EMSP are regularly confronted with critical mission events and adverse working conditions they cannot fully control. Therefore, active coping may be ineffective or possibly counterproductive in certain situations. Indeed, previous studies linked active coping to higher levels of stress and burnout in emergency workers (Cicognani et al., 2009; Prati et al., 2011).

It is proposed that *humor* enables EMSP to experience critical situations as less serious and threatening (Moran, 2002). Healthcare workers who used humor perceived work-related situations less stressful (Canestrari et al., 2021), and the use of humor was linked to less PTSD symptoms among firefighters (Sliter et al., 2014). However, humor is a very complex construct with various subtypes which may have opposite effects in handling stress (Leist & Müller, 2013; Martin et al., 2003). Indeed, humor was also associated with higher burnout levels in EMSP (Cicognani et al., 2009; Prati et al., 2011).

As an emotion-focused coping strategy, *religion* has been linked to less burnout symptoms (Boland et al., 2019) and higher levels of posttraumatic growth (Ogińska-Bulik & Zadworna-Cieślak, 2018), but also with more burnout symptoms and compassion fatigue in EMSP (Cicognani et al., 2009; Prati et al., 2011). In their concept of posttraumatic growth, Tedeschi and Calhoun (1996) assume increasing spirituality as an adaptive consequence of traumatic experiences. Accordingly, positive associations between stress symptoms and religious coping in EMSP could indicate emerging posttraumatic growth.

Moreover, EMSP reported to manage their work-related stress through *acceptance of negative emotions* as well as *positive reappraisal* (Boland et al., 2019; Kirby et al., 2011). *Acceptance* was consistently linked to increased posttraumatic growth (Kirby et al., 2011; Prati & Pietrantoni, 2009) and milder posttraumatic stress symptoms in EMSP (Zhao et al., 2020). *Positive reappraisal* was associated with more burnout and compassion fatigue symptoms (ALmutairi & El Mahalli, 2020; Cicognani et al., 2009) but was also related with stronger posttraumatic growth (Kirby et al., 2011).

## Adaptive Coping and Self-Efficacy

Self-efficacy refers to the deep conviction that one has sufficient resources and abilities to cope successfully with adversity (Bandura, 1997). Self-efficacy determines the individual’s approach and self-perception when coping with stressors. Thereby, it influences execution of coping strategies as well as the persistency of coping efforts (Bandura, 1997). As a result, self-efficacious individuals experience job stress less threatening, working conditions more positively, and focus more on available resources (e.g., social support) (Consiglio, Borgogni, Alessandri, & Schaufeli, 2013). Studies in the EMS found that personnel with longer work experience report higher self-efficacy, which contributed to less burnout and compassion fatigue as well as more compassion satisfaction (Cicognani et al., 2009; Groß et al., 2004; Prati et al., 2010). In nurses, the beneficial effect of self-efficacy on health and well-being was partially mediated through problem-focused coping (Chang & Edwards, 2015).

## Present Study

Coping behavior of EMSP may change with increasing professional experience and/or as a function of the recurrent exposure to stress and traumatic events (Essex & Scott, 2008). Through habituating with their work, EMSP will increasingly engage in coping strategies they experience as helpful in alleviating stress in the short-term (Figley, 2008). Resulting coping habits will conceivably differ from those of the general population as well as of occupations with other demands. Therefore, Cicognani et al. (2009) explored specific factors of coping strategies in 764 Italian emergency workers, including EMSP, firefighters, and civil-protection personnel. From the 14 coping strategies assessed with the Brief-COPE, an exploratory factor analysis extracted seven coping factors, i.e., *support/venting*, *active coping*, *positive reappraisal*, *humor*, *religion*, *self-distraction*, and *self-criticism*, which showed complex associations with the personnel’s quality of life and mental health.

The coping factor model identified by Cicognani et al. (2009) is yet to be confirmed. With this study, we tested whether Cicognani et al.’s factor model fits the coping behavior of German EMSP. Moreover, we hypothesized “maladaptive” coping (e.g., self-distraction, self-criticism) is linked to higher perceived stress, lower job satisfaction, and more mental and physical stress symptoms. Conversely, we expected “adaptive” coping (e.g., support/venting, active coping, positive reappraisal, humor, religion) to be linked to better health and well-being. Additionally, we hypothesized that EMSP with longer work experience show higher work-related self-efficacy. Higher self-efficacy was expected to correlate with higher job satisfaction, lower work-related stress, and fewer mental and physical symptoms.

## Method

### Procedure

The authors conducted an in-house training module offered seven times within three months at two ambulance stations of the local German Red Cross (GRC) division. Of the division’s 318 employees, 241 attended the training and were invited to participate in this study. Interested EMSP left their email address, and via email they received the link to the study survey. At the beginning of the survey, participants were informed about the study aims and procedures. A total of 115 employees declared their written informed consent and participated in the survey (46.6% response rate) that assessed sociodemographic characteristics (e.g., age, gender) and exposure to traumatic events, personality traits, mental and physical health conditions as well as coping strategies using standardized questionnaires. The survey also assessed other health-relevant factors such as emotion regulation and sense of coherence that were reported in previous studies (Behnke, Conrad, et al., 2019; Gärtner et al., 2019). The survey took approximately one hour for completion. Participants received no remuneration. The study protocol was approved by the Ulm University ethics committee.

### Participants

Regarding the variables investigated in this study, complete data were available from *N* = 106 EMSP (63.2% men), presenting 33.3% of the local GRC divisions’ total workforce. Participating EMSP were 18 to 61 years of age, *Mdn* (*IQR*) = 26 (15.8), and their work experience ranged from one month to 35 years, *Mdn* (*IQR*) = 3.3 (10.3) years. Additional sociodemographic characteristics are detailed in Table 1. Study participants corresponded well to the entirety of local EMS employees in terms of sex, stationing, and EMS work experience. Small differences occurred regarding employment type and age.

**Table 1:** Demographic Sample Characteristics Compared to the Local EMS Personnel

Demographic Variable	Study cohort		Local EMS employees		Statistical test		
	*n*	%	*n*	%	Test statistic	*p*	Effect size
**Total**	106	33.3^#^	318				
Sex					–	.229	-.061
Male	67	63.2	222	69.8			
Female	39	36.8	96	30.2			
Ambulance station					–	1	-.003
Ulm	74	69.8	223	70.1			
Heidenheim	32	30.2	95	29.9			
Employment form					χ^2^(2) = 11.51	.003	.165
Salaried	80	75.5	198	62.3			
Voluntary	16	15.1	101	31.8			
In apprentice	10	9.4	19	6.0			
Professional qualification							
EMT–paramedic (“Notfallsanitäter”)	64	60.4	–	–			
EMT–basic (“Rettungssanitäter”)	32	30.2	–	–			
EMT–paramedic trainee	10	9.4	–	–			
Family status							
Single	50	47.2	–	–			
Divorced	8	7.5	–	–			
Partnership/married	48	45.3	–	–			
	***M* (*SD*)**	***Mdn***	***M* (*SD*)**	***Mdn***			
**Age** **[years]**	29.8 (10.9)	26.0	32.1 (11.1)	27.5	*U* = 13906	.007	-.131
**EMS working experience [years]**	7.5 (8.7)	3.3	5.7 (5.5)	3.8	*U* = 16172.5	.629	-.023

*Note. ^#^*proportion of total staff. Population and sample frequency distributions were compared using Fisher’s exact tests and χ^2^ tests, where applicable, and φ as effect-size measure. Continuous variables were compared using Mann-Whitney *U*-tests using Cohen’s *r* as effect-size measure.

### Measures

*Coping strategies* were measured with the 28-item German Brief-COPE (Knoll et al., 2005). The Brief-COPE subscales’ internal consistency ranged from Cronbach’s α = .43–.89. As an exception, the subscale *behavioral disengagement* showed an inacceptable internal consistency of α = -.04 (see Supplementary Materials, Table X1, for details).

*Perceived work-related stress* was recorded with an EMS-specific questionnaire (Gärtner et al., 2019). On eight items, participants reported their perceived stress due to alarms, shift work, etc. on a 5-point Likert scale anchored at 0 (*never experienced*) and 4 (*very bothering*). Reponses were aggregated to a sum score (range: 0–32; Cronbach’s α = .77).

*Depressive symptoms* were measured with the 9-item German Patient Health Questionnaire scale for depression (PHQ-9; Löwe et al., 2002). Responses are recorded on a four-point Likert scale ranging from 0 (*not at all*) to 3 (*almost every day*) and were aggregated to a sum score (range: 0–27; Cronbach’s α = .83).

*Posttraumatic symptoms* were assessed with the German PTSD Checklist for DSM-5 (PCL-5; Krüger-Gottschalk et al., 2017). Participants were requested to recall their most stressful life event. As previously reported, 53% of the EMSP participating in this study encountered their most stressful life events in the line of their duty (Behnke, Rojas, et al., 2019). With eight qualitative items, the PCL-5 evaluates whether the most stressful life event fulfils the DSM-5 criteria of a traumatic event. On 20 items, participants rated the severity of their posttraumatic stress symptoms on a 5-point Likert scale ranging from 0 (*not at all*) to 4 (*very strong*). Severity ratings were aggregated to a sum score (range: 0–80, Cronbach’s α = .91).

*Physical ailments* were assessed using the 15-item German Patient Health Questionnaire scale for physical symptoms (PHQ-15; Löwe et al., 2002). The item asking for menstrual pain was excluded for reasons of gender comparability. Responses are recorded on a 3-point Likert scale ranging from 0 (*not at all*) to 2 (*very strong*). The sum score of all items represents the severity of physical ailments (range: 0–30, Cronbach’s α = .84).

*Job satisfaction* was evaluated using a subscale of the German Michigan Organizational Assessment Questionnaire (Cammann et al., 1979). On three items, participants rated their job satisfaction on a 4-point Likert-scale ranging from 1 (*strongly disagree*) to 4 (*strongly agree*). Responses were combined as sum score (range: 3–12, Cronbach’s α = .69).

*Work-related self-efficacy* was assessed using the two items of the Professional Self-efficacy Expectation Scale with the highest item-total correlation (Schyns & Collani, 2014). Responses were recorded on a 4-point Likert scale ranging from 0 (*not at all*) to 4 (*very strong*) and combined to a sum score (range: 0–8, Cronbach’s α = .67).

### Statistical Analyses

Statistical analyses were performed in R 3.6.2 (R Core Team, 2019). To examine whether the factor structure reported in Cicognani et al. (2009) fits the present data, a confirmatory factor analysis (CFA) was performed using the *lavaan* package (Rosseel, 2012). As a majority of the Brief-COPE items did not follow uni- or multivariate normal distribution (Energy test: *E* = 2.44, *p* < .001), we used pairwise maximum likelihood (PML) estimators as a computationally less intense alternative to full information maximum likelihood (FIML) (Katsikatsou et al., 2012). The absolute χ^2^ statistic and its *p*-value (*p* > .05), the root mean square error of approximation (RMSEA ≤ .06) and its 90% confidence interval (CI), and robust versions of the standardized root mean square residual (SRMR ≤ .08), the comparative fit index (CFI ≥ .95), and the Tucker-Lewis index (TLI ≥ .95) were used as model fit criteria (Hu & Bentler, 1999). Convergent and discriminant factor validity was examined applying the criteria by Fornell and Larcker (1981), and Bollen’s ω (Raykov, 2001) quantified the internal factor consistency. Bivariate correlations were analyzed using nonparametric Spearman correlations because several variables were not normally distributed. *p*-Values were corrected for multiple testing using the false discovery rate (FDR) (Benjamini & Yekutieli, 2001).

## Results

### Confirmatory Factor Analyses

All Brief-COPE subscales were non-normal distributed, and some subscales were strongly right-skewed, that is, these strategies were almost never used by our study cohort (Table X1, Supplementary Materials). This was also observed by Cicognani et al. (2009), and in accordance with their procedure, we disregarded the items 3/8 (denial: skew = 2.32), 6/16 (behavioral disengagement: skew = 1.45), and 4/11 (substance use: skew = 2.10) in the CFA. Additionally, the scales self-blame (skew = 1.27) and religion (skew = 1.87) displayed a strong right skew in our sample. We nevertheless retained these items to allow testing the adequacy of Cicognani et al.’s factor model in our data.

**Figure 1:**
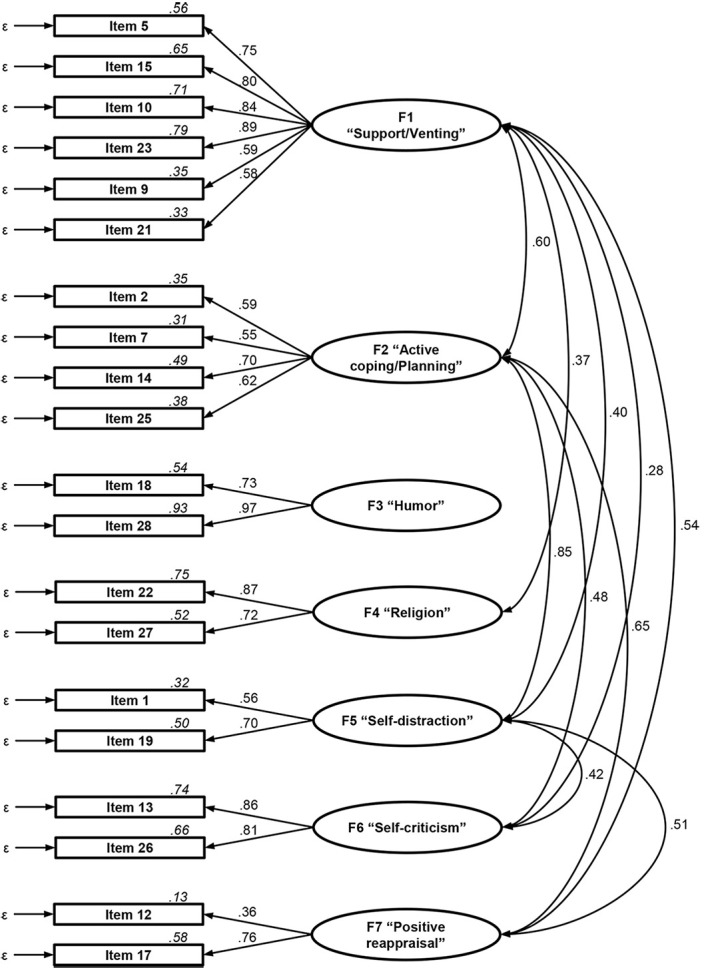
Results of the Confirmatory Factor Analysis Examining the Fit of Cicognani et al.’s (2009) Seven-Factor Model of Coping to the Data of this Study *Note. N* = 106. Values on paths indicate standardized regression coefficients (β) and values on covariance paths indicate significant factor correlations (*r*). Italic values above the items display the explained variance per item (*R*^2^).

The CFA revealed the model by Cicognani et al. (2009) fits our data relatively well: robust-χ^2^(5.54) = 9.47, *p* = .120; CFI_rob_ = .926; TLI_rob_ = .911; SRMR_rob_ = .069; RMSEA < .001, 90% CI [.001, .041], *p*_RMSEA_ = .988. The first factor (Figure 1) comprised the six items of the subscales *Emotional support, Instrumental support,* and *Venting* (standardized factor loadings: β = .58–.89, *p*’s < .001) with an internal factor consistency of ω = .89.

The second factor comprised the items of *Active coping* and *Planning* (β = .55–.70, *p*’s < .001; ω = .71). The third factor presented the *Humor* subscale (β = .73–.97, *p*’s < .001; ω = .83), the fourth *Religion* (β = .72–.87, *p*’s < .001; ω = .78), the fifth *Self-distraction* (β = .56–.70, *p*’s < .001; ω = .58), the sixth *Self-criticism* (β = .81–.86, *p*’s < .001; ω = .83), and the seventh *Positive reappraisal* (β = .36–.76, *p*’s < .005; ω = .49).

Examining the factors’ convergent and discriminant validity (Table 2) revealed that support/venting, humor, religion, and self-criticism are clearly distinguishable albeit correlated factors. Conversely, the items of active coping/planning share considerable variance with the items of self-distraction and positive reappraisal, indicating that their factors are not clearly separable. As a result, these factors had a low internal factor consistency (see Table 2).

**Table 2:** Indicators of Internal Factor Consistency ω (at Diagonal), Convergent and Discriminant Validity Along With Factor Correlations

Coping Factor	F1	F2^†^	F3	F4	F5^†^	F6	F7^†^
F1 Support/Venting	**.89**	.60***	-.17	.37***	.40*	.28*	.54***
F2 Active coping/planning		**.71**	-.04	.19	.85***	.48***	.65***
F3 Humor			**.83**	-.16	.22	-.11	.19
F4 Religion				**.78**	.02	.06	.28
F5 Self-distraction					**.58**	.42**	.51**
F6 Self-criticism						**.83**	.16
F7 Positive reappraisal							**.49**
Average variance extracted (AVE)	.576	.380	.709	.650	.420	.705	.354
Maximum shared variance (MSV)	.356	.724	.047	.139	.724	.226	.422

*Note.* An average variance extracted of AVE > .50 indicates sufficient convergent factor validity (i.e., more than 50% of the items’ variances converged on their common factor). Satisfactory discriminant factor validity is assumed when the maximum shared variance MSV < AVE. Factors indicated with ^†^ violate aforementioned criteria.

*p < .05. **p < .01. ***p < .001, two-tailed, corrected for multiple testing with FDR.

### Correlation of Coping Factors With Well-Being and Health

Correlation analyses (Table 3) indicated that *support/venting* was less used by older EMSP, whereas no associations emerged with other studies variables. *Active coping/planning, religion, self-distraction,* and *positive reappraisal* were not related to any study variable. In trend, EMSP with more work experience also reported more *self-criticism* (*p*_FDR_ = .102), and frequent use of *self-criticism* was positively associated with higher perceived stress, more mental and physical symptoms, and lower job satisfaction.

**Table 3:** Spearman Rank Correlations (N = 106)

Coping Factor	Age	Sex^a^	EMS work experience	PCL-5	PHQ-15	PHQ-9	Perceived Stress	Job Satisfaction^b^	Work-related self-efficacy
F1 Support/Venting	-.28*	-.07	-.08	.09	-.07	-.15	.07	.17	.14
F2 Active coping/Planning	.05	.05	.05	.23	.05	.02	.16	-.07	.11
F3 Humor	.15	.24	.11	-.01	.08	.09	-.03	.00	.34*
F4 Religion	-.10	-.10	.00	.26	.04	.00	.13	.10	-.08
F5 Self-distraction	.11	.13	.10	.16	.01	.01	.10	-.05	.26
F6 Self-criticism	.09	-.02	.22	.49***	.32*	.34*	.27*	-.27*	-.22
F7 Positive reappraisal	-.10	-.02	-.10	.17	-.02	-.05	.09	.12	.13
Work-related self-efficacy	.21	.29*	.18	-.22	-.23	-.26	-.04	.24	

^a^Positive coefficient indicate higher values in men than women. ^b^two missing values.

*p < .050. ***p < .001, two-tailed, corrected for multiple testing with FDR.

These associations were also supported by the zero-order correlations between the Brief-COPE subscales and the study variables (Table X2, Supplementary Materials). Additionally, we observed relevant correlations of the Brief-COPE’s acceptance subscale, which has been neglected in the CFA in order to test the factor solution reported by Cicognani et al. (2009). In detail, EMSP in our sample who reported higher *acceptance* showed less stress-related symptoms (PCL-5: *r*_S_ = -.21, *p*_FDR_ = .138; PHQ-15: *r*_S_ = -.31, *p*_FDR_ = .020; PHQ-9: *r*_S_ = -.32, *p*_FDR_ = .018).

### Work-Related Self-Efficacy and Coping

Male (*p*_FDR_ = .037) and older EMSP (*p*_FDR_ = .102) reported higher work-related self-efficacy, which was associated in trend with higher job satisfaction (*p*_FDR_ = .081) and less posttraumatic (*p*_FDR_ = .101), depressive (*p*_FDR_ = .053), and physical stress symptoms (*p*_FDR_ = .090, cf. Table 3). Moreover, self-efficacy correlated with a conceivably more adaptive coping behavior, in a way that EMSP with higher self-efficacy were prone to use less *self-criticism* in trend (*p*_FDR_ = .102) as well as more *humor* (see Table 3) and *acceptance* (*r*_S_ = .38, *p*_FDR_ = .002; Table X2, Supplementary Materials).

## Discussion

We investigated habitual coping behavior in a cohort of German EMSP and its relevance for the personnel’s health and well-being. Thereby, we replicated the seven-factor structure of Brief-COPE items which has been previously identified by Cicognani et al. (2009) in Italian emergency workers. Among these coping factors, *self-criticism* showed significant associations with stress, job satisfaction, and stress symptoms of EMSP.

Similar to the Italian emergency workers (Cicognani et al., 2009), our cohort of German EMSP rarely engaged in *denial*, *behavioral disengagement*, and *substance (ab)use* when coping with stress. Unlike the Italian sample, however, our cohort of EMSP almost never coped through *religion*. Cross-cultural studies indicate that reliance on religion in coping with adversity and stress varies across countries (Chai et al., 2012; Shirazi et al., 2011). Therefore, differences in the use of coping strategies between our study cohort and that of Cicognani et al. (2009) may result from cultural differences between Italian and German rescue personnel. Future cross-cultural research should compare coping in frontline workers with different cultural and social background.

Consistent with Cicognani et al. (2009), our CFA corroborated a factor unifying items of *support seeking* and *venting*, indicating that EMSP seek the support of others to share their unpleasant emotions and find comfort. Unexpectedly, this factor was not associated with better health or well-being, adding to previous inconsistent findings on the adaptiveness of social support for the well-being of EMSP (Boland et al., 2019; Essex & Scott, 2008; Feldman et al., 2021; Fjeldheim et al., 2014; Kleim & Westphal, 2011; Kshtriya et al., 2020; Wild et al., 2016). One reason for these heterogeneous findings could be the timing of social support: In their review, Wagner at al. (2016) conclude that pre-trauma social support can enhance resilience against PTSD, while post-trauma social support appears to promote posttraumatic growth. Conceivably, EMSP actively seek social support when feeling particularly stressed, and this adaptive behavior could enable personal growth. Moreover, previous research has differently defined and operationalized social support: While we included support and venting into one factor (cf. Cicognani et al., 2009), other studies focused on received and/or perceived social support by different groups, e.g., family, colleagues (Fjeldheim et al., 2014; Wild et al., 2016).

As previously reported (Essex & Scott, 2008), we found that older EMSP reported less support seeking and a lower tendency to communicate their feelings. Senior EMSP with many years of work experience are likely to have encountered more traumatic mission events, and studies showed that after highly aversive missions, a relevant proportion of EMSP refrains from talking to their colleagues to avoid showing personal weakness, possible consequences of perceived mistakes, and “unnecessarily” raising their colleagues’ emotional burden (Häller et al., 2009; Richter, 2014). This behavior could lead to social distancing and isolation in the long-term. However, in Western societies, there is a general trend toward decreasing social support networks across the lifespan (Nicolaisen & Thorsen, 2017), and social isolation particularly affects men (e.g., Gurung, Taylor, & Seeman, 2003; Walen & Lachman, 2000). In our cohort, the correlation of higher age and work experience with decreased social support/venting could be specifically pronounced, as the EMS has been primarily a “male profession”, and our study participants with longer work experience were almost exclusively men. Preventive measures to maintain EMSPs’ health could aim to impart social and emotional competencies among colleagues and supervisors, establish an institutional support culture, and develop structured professional counselling interventions for personnel (Wild et al., 2020).

In this sample, using *humor* as a coping strategy was not associated with well-being and health. Previous evidence on humor in helping profession is mixed. Some studies showed, humor allowed perceiving work less stressful (Canestrari et al., 2021) and was linked to fewer PTSD symptoms (Sliter et al., 2014). Other studies linked humor to higher burnout symptoms (Cicognani et al., 2009; Prati et al., 2011). This inconsistency may originate from different styles of humor which may exert opposite effects in stress coping (Leist & Müller, 2013). Black or “gallows” humor presents a form of emotional avoidance that can help EMSP to quickly distance from adverse experiences (Moran, 2002). However, in the long-term, black humor may establish cynicism towards their patients in EMSP, and this attitude might compromise the emotional support they receive from their family and friends (Rowe & Regehr, 2010). In this study and previous studies (Cicognani et al., 2009; Prati et al., 2011), humor was assessed with two items, thus not allowing to differentiate humor styles. Future studies are required to investigate the role of humor styles more comprehensively to understand its effect on the health and well-being of EMSP.

In our study, the factors *active coping/planning* and *positive reappraisal* were unrelated to EMSPs’ well-being and health, whereas previous studies linked *active coping* to reduced stress (Brown et al., 2002; Jamal et al., 2017; Prati et al., 2011) and fewer stress symptoms (Kirby et al., 2011). Moreover, the inclination to find positive reinterpretations of adverse experiences has been linked to stronger posttraumatic growth (Kirby et al., 2011). In our study, however, the factors overlapped with the EMSPs’ engagement in *self-distraction*. This suggests that EMSP tend to actively engage in compensatory activities and denying stress through positive reinterpretations *in order to* distract themselves from work-related stress.

Unlike the classical view of active coping and positive reappraisal as adaptive stress coping, in EMSP, such attempts rather reflect a *distraction* tendency to achieve short-term stress relief. In par with this, Levy-Gigi et al. (2016) reported firefighters engage in distractive strategies to achieve immediate stress relief, although such distractive coping attempts exert counterproductive effects on the regulation of stress in the long-run (Cicognani et al., 2009; Kirby et al., 2011; LeBlanc et al., 2011). However, in our study, using these strategies seemed to have no implications for the EMSPs’ health status and well-being. Additional research is required to better distinguish the short- or long-term motives of frontline workers to engage in distractive coping strategies.

In addition, active coping aims to overcome a stressful situation through planning and problem solving. Thus, the actual effectiveness of this strategy depends on whether stressors are actually controllable and changeable (Folkman & Moskowitz, 2004). As EMSP regularly face adverse situations which they may not be able to control or change, it could be that attempting to actively change uncontrollable problems has no (Gärtner et al., 2019) or even opposite implications for the well-being of EMSP (Cicognani et al., 2009; Prati et al., 2011). Persistent attempts to find solutions for uncontrollable adversity might even initiate rumination (Ayduk & Kross, 2010), which is a major risk factor for developing PTSD, depression, and burnout in EMSP and firefighters (e.g., Bryant & Guthrie, 2007; Gärtner et al., 2019; Wild et al., 2016).

Correspondingly, our results indicate that engaging in *self-critical* reflections about one’s actions and feelings is associated with poorer health and well-being in EMSP. This result corroborates previous studies implicating self-criticism as a maladaptive coping strategy (Boland et al., 2019; Boudreaux et al., 1997; Cicognani et al., 2009; Kirby et al., 2011; Prati et al., 2011). Self-criticism involves repetitive negative evaluations of one’s own abilities and decisions. In this sense, it is closely related to rumination as the tendency to repeatedly focus mentally on negative emotional experiences as well as their causes and consequences (James et al., 2015). Longitudinal studies are warranted to assess self-criticism and rumination in the prospect of health and well-being in EMSP.

Beyond the coping factors reported by Cicognani et al. (2009), the BriefCOPE subscale *acceptance* was linked to higher self-efficacy and better well-being in EMSP. This result suits previous findings and meta-analyses which established acceptance as highly adaptive in retaining health upon adverse experiences (Aldao et al., 2010; Kirby et al., 2011; Schäfer et al., 2017; Zhao et al., 2020). Acceptance-related elements are featured in several evidence-based therapeutic approaches (e.g., Mentalization-based therapy, Bateman & Fonagy, 2012; Acceptance and commitment therapy, Hayes, 2016), and initial research on stress-preventive trainings in EMSP indicates that imparting strategies to differentiate, name, and accept unpleasant feelings can decrease symptoms of burnout and emotional exhaustion (Buruck & Dörfel, 2018).

Bandura (1997) theorized self-efficacy enhances stress resilience through influencing which and how persistently coping strategies are executed upon stress. Accordingly, self-efficacy was positively linked to problem-focused and active coping and negatively linked to emotion-focused coping in nurses (Chang & Edwards, 2015). Our findings partially corroborate this perspective, as we found EMSP with higher self-efficacy to use less *self-criticism* when coping with stress. However, self-efficacy was not linked to strategies such as *coping/planning* or *support/venting*. Instead, it was linked to *acceptance* and *humor* presenting rather emotion-focused coping strategies. Moreover, in line with previous studies in the EMS (Behnke, Conrad, et al., 2019; Cicognani et al., 2009; Groß et al., 2004; Prati et al., 2010, 2011), personnel with longer work experience reported higher self-efficacy, and higher self-efficacy was associated with higher job satisfaction and fewer physical and depressive symptoms in trend. Future research could aim to comprehensively examine the nature and relationship of self-efficacy, acceptance, humor, and self-criticism/rumination with health and well-being in frontline workers.

### Limitations and Future Directions

Studies did not conclude on a unique hierarchical structure of the coping strategies assessed with the Brief-COPE (Hanfstingl et al., 2021; Solberg et al., 2021). Therefore, we decided to test the adequacy of the factor solution explored by Cicognani et al. (2009) and were able to replicate the factor structure. However, additional reliability analyses showed that some of the extracted factors overlap, which compromises their factor reliability. Our sample size is rather small for conducting CFA, and future studies should aim to recruit larger samples. Moreover, simulation studies demonstrated that drawing reliable conclusions about model-to-data fit in CFA is not trivial, as Hu and Bentler’s (1999) criteria may lead to unreliable results (Beierl et al., 2018; Heene et al., 2011).

Compared to previous studies in the EMS, the response rate in our study (46.6%) is in the upper range (Brown et al., 2002; Fritz & Sonnentag, 2005). Nevertheless, generalizability of our findings is limited by convenience sampling. Results may be biased by differences between study participants and non-participants; i.e., EMSP with more stress symptoms and/or socially inappropriate coping behaviors (e.g., substance abuse) were perhaps unmotivated or avoided participation (*non-response bias*; Bortz & Döring, 2004). EMSP who were unable to work or had changed their profession due to severe stress-related health problems could not be included in the study. This may lead to biased results, as highly stressed personnel might use less effective coping strategies (*healthy-worker effect*; Costa, 2003). Future studies should compare coping habits of EMSP capable to work and those with work-related health problems.

Limitations in validity could result from *retrospective recall errors* (Jonkisz et al., 2012). That is, EMSP remembered stressful events but did not associate them with specific coping strategies, or they are completely unaware of using certain strategies. Moreover, the study’s cross-sectional correlative design does not allow causal or predictive conclusions. Longitudinal research is required to better characterize the interplay of coping, stress exposure, and well-being through high-frequency measurements, for example, on a daily basis using mobile phone applications. Such “ecological momentary assessments” enable identifying coping behaviors with prospective relevance in handling daily occupational stressors and traumatic mission events in the EMS.

### Conclusions

Effective coping with occupational stressors is pivotal for retaining health and well-being in emergency workers. With this cross-sectional study in German EMSP, we confirmed seven coping factors that were previously identified by Cicognani et al. (2009) in Italian emergency workers. Among these coping factors, only *self-criticism* was significantly associated with the EMSPs’ work-related stress, job satisfaction, and well-being. Additionally, exploratory correlations indicated that using *acceptance* was potentially beneficial for the self-efficacy and well-being of EMSP. Our findings implicate investigating the use and relevance of self-criticism and acceptance in prospective longitudinal designs. Determining the relevance of certain coping strategies regarding health and well-being is key to developing occupation-tailored preventive interventions.

## Supplementary Materials

Supplementary tables presenting: Descriptive statistics, internal consistencies, and univariate normality assessment of Brief-COPE subscales (Table X1), and Spearman correlations between Brief-COPE subscales and the other study variables (Table X2) (for access see Index of Supplementary Materials below).

10.23668/psycharchives.5585 Supplement 1 Supplementary materials to "Coping in the Emergency Medical Services: Associations with the personnel’s stress, self-efficacy,job satisfaction, and health"

RojasR.
HickmannM.
WolfS.
KolassaI.-T.
BehnkeA.
 (2022). Supplementary materials to
"Coping in the Emergency Medical Services: Associations with the
personnel’s stress, self-efficacy, job satisfaction, and
health"
[Additional information]. PsychOpen. 10.23668/psycharchives.5585
PMC966734136397746

## Data Availability

The datasets for this manuscript are not publicly available because we do not have the consent of the ethics committee or our participants to grant any form of access to or insight in all or parts of the collected data.
