# Supplementary material for: Coping in the Emergency Medical Services: Associations With the Personnel’s Stress, Self-Efficacy, Job Satisfaction, and Health
Source: Clin Psychol Eur. 2022 Mar 31;4(1):e6133. doi: 10.32872/cpe.6133 (PMC9667341; doi:10.32872/cpe.6133)
Supplement: Supplement 1 [file cpe-04-6133-s01.pdf]

SUPPLEMENTARY MATERIAL

to the manuscript

Coping in the Emergency Medical Services: Associations with the Personnel's Stress, Self-Efficacy, Job Satisfaction, and Health

published in *Clinical Psychology in Europe*

https://doi.org/10.32872/cpe.6133

Roberto Rojas[a*], Maxi Hickmann[a], Svenja Wolf[a], Iris-Tatjana Kolassa[b], Alexander Behnke[b]

[a] University Psychotherapeutic Outpatient Clinic, Institute of Psychology and Education, Ulm University, Ulm, Germany

[b] Clinical and Biological Psychology, Institute of Psychology and Education, Ulm University, Ulm, Germany

[*] Corresponding Author: Roberto Rojas, University Psychotherapeutic Outpatient Clinic, Institute of Psychology and Education, Ulm University, Schaffnerstraße 3, 89073 Ulm, Germany. E-mail: roberto.rojas@uni-ulm.de, Phone: +49/731-50 31601, Fax: +49/731-50 1231601

Supplementary Table X1. *Descriptive statistics, internal consistencies, and univariate normality assessment of Brief-COPE subscales*

**Brief-COPE subscale**	**Descriptive statistics**								**Anderson-Darling test**	
	M	*SD*	*Med*	*Min*	*Max*	Skew	Kurtosis	Cronbach's α	*A*	*p*
Self-distraction	2.88	1.74	3	0	6	-0.01	-1.04	.57	1.99	<0.001
Denial	0.31	0.72	0	0	3	2.32	4.53	.67	25.22	<0.001
Emotional support	3.36	2.04	4	0	6	-0.29	-1.27	.83	3.29	<0.001
Behavioral disengagement	0.66	1.02	0	0	4	1.45	0.97	-.04	14.59	<0.001
Positive reframing	2.82	1.39	3	0	6	-0.02	-0.31	.43	2.34	<0.001
Humor	1.95	1.77	2	0	6	0.69	-0.55	.83	3.91	<0.001
Active Coping	2.36	1.66	2	0	6	0.30	-0.78	.60	2.47	<0.001
Substance (ab)use	0.53	1.09	0	0	5	2.10	3.63	.88	21.52	<0.001
Instrumental support	2.57	1.98	2	0	6	0.42	-1.01	.89	3.04	<0.001
Venting	1.98	1.70	2	0	6	0.46	-0.84	.65	3.54	<0.001
Planning	2.75	1.75	3	0	6	0.09	-0.92	.59	1.86	<0.001
Acceptance	4.15	1.54	4	0	6	-0.58	-0.35	.65	3.31	<0.001
Self-blame	1.44	1.63	1	0	6	1.27	0.90	.82	6.78	<0.001
Religion	0.85	1.50	0	0	6	1.87	2.91	.77	16.53	<0.001

Supplementary Table X2. *Spearman correlations between Brief-COPE subscales and the other study variables*

**Brief-COPE subscale**	**Age**	**Sex**	**EMS work experience**	**PCL-5**	**PHQ-15**	**PHQ-9**	**Perceived stress**	**Job satisfaction**	**Work-related self-efficacy**
Self-distraction	.13	.10	.15	.13	-.04	-.04	.02	-.04	**.30***
Denial	.13	.00	.17	.26	.25	.23	.14	-.11	-.04
Emotional support	**-.32***	-.03	-.11	.05	-.09	-.16	.01	.18	.17
Behavioral disengagement	.07	-.04	.13	.12	.02	.05	-.03	.10	.08
Positive reframing	-.04	-.02	-.08	.05	-.15	-.21	-.04	.20	.22
Humor	.12	**.21**	.10	-.01	.09	.10	-.03	-.05	**.34****
Active Coping	.03	.09	-.02	.11	.03	.00	.09	-.02	.17
Substance (ab)use	.05	.02	.21	**.28***	.22	.24	.07	-.01	.00
Instrumental support	-.23	-.07	-.04	.08	-.03	-.11	.04	.14	.11
Venting	-.14	-.05	-.06	-.06	-.14	-.20	.15	.12	.19
Planning	.16	.18	.11	**.28***	.13	.12	.24	-.16	.02
Acceptance	-.10	.14	-.01	-.21	**-.31***	**-.32***	-.15	.14	**.38****
Self-blame	.08	-.07	.23	**.51*****	**.36****	**.38****	.26	-.27	**-.29***
Religion	.08	-.04	.10	**.31***	.09	.09	.08	.08	-.02

Note: $N = 106$, * $p < .050$, ** $p < .010$, *** $p < .001$, two-tailed, corrected for multiple testing with FDR. Note that ‘Behavioral disengagement’ should not the interpreted due to its insufficient internal consistency (Cronbach’s α = -.04).

Supplementary References

Cammann, C., Fichman, M., Jenkins, D., & Klesh, J. (1979). *The Michigan Organizational Assessment Questionnaire: Unpublished Manuscript.*

Gärtner, A., Behnke, A., Conrad, D., Kolassa, I.-T., & Rojas, R. (2019). Emotion Regulation in Rescue Workers: Differential Relationship With Perceived Work-Related Stress and Stress-Related Symptoms. *Frontiers in Psychology*, *9*, 2744. https://doi.org/10.3389/fpsyg.2018.02744

Knoll, N., Rieckmann, N., & Schwarzer, R. (2005). Coping as a mediator between personality and stress outcomes: A longitudinal study with cataract surgery patients. *European Journal of Personality*, *19*(3), 229–247. https://doi.org/10.1002/per.546

Krüger-Gottschalk, A., Knaevelsrud, C., Rau, H., Dyer, A., Schäfer, I., Schellong, J., & Ehring, T. (2017). The German version of the Posttraumatic Stress Disorder Checklist for DSM-5 (PCL-5): Psychometric properties and diagnostic utility. *BMC Psychiatry*, *17*(1), 379. https://doi.org/10.1186/s12888-017-1541-6

Löwe, B., Spitzer, R., Zipfel, S., & Herzog, W. (2002). *Gesundheitsfragebogen für Patienten (PHQ-D). Komplettversion und Kurzform. Testmappe mit Manual, Fragebögen, Schablonen.* Pfizer.

Schyns, B., & Collani, G. (2014). Berufliche Selbstwirksamkeitserwartung. Zusammenstellung sozialwissenschaftlicher Items und Skalen. *Zusammenstellung sozialwissenschaftlicher Items und Skalen (ZIS)*. https://doi.org/10.6102/ZIS16
